# Prevalence of Depression and Post-Traumatic Stress Disorder (PTSD) Symptoms among Orthopaedic Post-Trauma Patients in Hospital Tuanku Jaafar Seremban (HTJS)

**DOI:** 10.5704/MOJ.2407.005

**Published:** 2024-07

**Authors:** ZF Zairul-Nizam, WE Thye, VSH Ng, CFG Soh, VJW Tan

**Affiliations:** Department of Orthopaedics, International Medical University, Seremban, Malaysiaaff

**Keywords:** orthopaedic post-trauma, depression symptoms, PTSD symptoms

## Abstract

**Introduction:**

Post-trauma patients are at risk of developing symptoms of post-traumatic stress disorder (PTSD) and major depression. The primary goal of this study is to estimate the prevalence of PTSD and depression symptoms in patients who have been hospitalised for the treatment of physical trauma. Additionally, we wanted to compare the prevalence of PTSD or depression symptoms alone versus PTSD associated with depression symptoms, in orthopaedic post-trauma patients.

**Materials and Methods:**

This study had involved orthopaedic post-trauma patients in the orthopaedic ward and clinic of Hospital Tuanku Jaafar (HTJ), Seremban, Malaysia, using an online questionnaire, which consist of English and Malay language. We then determined the prevalence of depression and PTSD symptoms in orthopaedic post-trauma patients and compared this prevalence to the severity of the injuries sustained and any association between PTSD and depression symptoms.

**Results:**

Only 12.9% of the participants are likely to have post-traumatic stress disorder (PTSD) symptoms and 43.3% of participants have depression symptoms. There is no significant association between patient demographics and severity of the injuries with the prevalence of post-traumatic stress disorder (PTSD) and depression symptoms. However, of those deemed likely to have PTSD, 93.5% of them had both post-traumatic stress disorder (PTSD) symptoms as well as depressive symptoms.

**Conclusion:**

Only a few of the participants are likely to develop post-traumatic stress disorder (PTSD) while almost half of the participants are likely to have developed depression. Physicians caring for trauma patients should screen them for early symptoms of PTSD and depression and treat them accordingly.

## Introduction

According to the 4th report released by the National Trauma Database (NTrD) of Malaysia, a total of 166,768 trauma patients were admitted into the Emergency Departments in the 8 participating hospitals of the NTrD in Malaysia in 2009^1^. In addition to the physical injuries sustained from trauma, these patients are at an elevated risk for psychiatric disorders such as post-traumatic stress disorder (PTSD) and major depression which is believed to be one of the most common comorbid disorders associated with PTSD^2^. The development of these mental illnesses following traumatic injuries is associated with unfavourable health outcomes such as a reduction in self-assessed quality of life and to some extent, physical functioning and impairment^3^. These potential psychiatric disorders may hinder a patient’s complete recovery following trauma. Based on available literature, depression and PTSD are prevalent psychiatric disorders that may occur in post-trauma orthopaedic patients. In a 2018 study, 500 adults with an injury score of more than 10 who were admitted to a Level 1 trauma centre, with 64.8% of them being screened positive for depression within one year after their injury, and another 44.4% of patients screened positive for PTSD^4^. In another prospective cohort study with a 2-year follow-up period, 99 participants with severe multiple traumas were assessed, with results showing that 20% of the participants had PTSD and another 27% had depression at 2 years post injury^5^.

PTSD is a complex psychiatric disorder and a disabling consequence of traumatic injury. The PTSD Checklist (PCL-5), a widely validated self-report measure, is a 20-item measure that assesses the Diagnostic and Statistical Manual of Mental Disorders 5 (DSM-5) symptoms of PTSD. It is used not only as a screen, but also to make a provisional PTSD diagnosis and monitor symptom changes throughout treatment^6^. According to DSM-5, one qualifies for the diagnosis of PTSD only after experiencing trauma and exhibiting several symptom criteria for more than one month. If symptoms last for less than one month, the diagnosis becomes that of an acute stress disorder (ASD)^7^. In a 2020 study, 452 adult trauma patients participated in a PCL-5 survey in a post-hospital outpatient clinic visit and were screened for PTSD. Results showed that 26% of the trauma patients screened positive: indicating that the prevalence of PTSD among post trauma patients far exceeds the general population where the prevalence is approximately 6^8^.

Risk factors for screening positive for PTSD were also measured in this study – results showed that pedestrians who were hit by motor vehicles and victims of crime were more likely to develop PTSD, whilst the injury severity score (ISS) and types of injury sustained were not associated with a positive screening^8^.

As PTSD is prevalent among post orthopaedic trauma patients, it is important to identify the risk factors so that acute intervention such as pharmacotherapy or psychotherapy can be initiated^8^. For example, being a woman and being younger were frequently associated with post-traumatic stress symptoms (PTSS), and thus are more likely to develop PTSD^5^. Additionally, the use of avoidant coping strategies, poorer mental health and cognitive functioning were also significant predictors for PTSS. Since patients that use avoidant coping strategies are more prone to develop PTSS, healthcare professionals should promote patient participation and the use of functional coping strategies in the rehabilitation process to enhance self-efficacy in severely injured patients^5^.

Yang *et al* in 2020 identified the prevalence and risk factors associated with underlying depression in post-orthopaedic trauma patients^9^. Their study revealed a positive association between post traumatic pain and depression. The explanation that was given is that patients are more likely to develop catastrophic thinking and become depressed when in pain, which worsens the condition and leads to a vicious cycle. Therefore, it is important to actively manage pain in post-traumatic orthopaedic patients to reduce their anxiety and depression. The same study also reported that unmarried patients had a higher risk of depression. Unmarried patients tend to have negative emotions as they lack family support which results in psychological isolation. Good social support is beneficial to the improvement of a patient's negative emotions hence reducing the risk of developing depression^9^. Our primary goals are to estimate the prevalence of PTSD and depression in patients who have been hospitalized for the treatment of physical injuries sustained from various types of traumas, as well as assess their knowledge in the aforementioned mental disorders. Furthermore, we were interested to evaluate any association between depression and PTSD symptoms and the different levels of trauma severity experienced by these patients, measured by the Injury Severity Score (ISS).

## Materials and Methods

We sought to determine the prevalence of depression and PTSD symptoms in orthopaedic post-trauma patients in Hospital Tuanku Jaafar (HTJ), Seremban, Malaysia, a tertiary trauma centre with an advanced trauma service. Specifically, we wanted to evaluate the prevalence of depression and PTSD symptoms between patients with different severity (ISS scores of less than 15 and more than 15), and to compare the prevalence of PTSD or depression symptoms only with PTSD associated with depression symptoms in orthopaedic post-trauma patients.

A cross-sectional, prospective study was conducted in the orthopaedic wards and clinics of Hospital Tuanku Jaafar (HTJ). Patients aged between 18–50 were eligible for inclusion in the study, except for those with known mental illnesses such as depression, schizophrenia, bipolar disease, etc. Those with chronic neurological diseases such as Parkinson’s Disease, Alzheimer’s Disease and Dementia or chronic ongoing illnesses such as cancer, Human Immunodeficiency Virus (HIV) and chronic kidney disease CKD were similarly excluded^10^. Participants were followed-up between November 2022 to June 2023.

A structured interviewer assisted questionnaire, available in both English and Bahasa Malaysia had been constructed through Google Form, the link of which was then administered to participants. Patient privacy and confidentiality had been assured prior to the interview. The questionnaire consists of four sections: sociodemographic, characteristics of the trauma and two screening tools for PTSD and depression symptoms. The mean time to complete the questionnaire was approximately 10 minutes.

The PTSD Checklist (PCL-5) questionnaire requires the participants to rate the degree to which they were bothered by the symptoms in the past week on a scale of 0 (not at all) to 4 (extremely). The cut-off score for likely PTSD is 3311.

The Eight-Item Patient Health Questionnaire Depression Scale (PHQ-8) questionnaire is used as a screening tool for depression among patients. This questionnaire is widely used and is known as a valid diagnostic tool which evaluates the severity of depression among patients. It consists of eight items determining the frequency of depressive symptoms which were experienced in the past two weeks. Participants are required to rate them on the scale of 0 (not at all) to 3 (nearly every day). Scores of 5, 10, 15, and 20 represent the cut-off points for mild, moderate, moderately severe and severe depression, respectively^11^.

All collected data were then transported into the IBM Statistical Package for Social Science (SPSS) version 28.0.1 for analysis. Descriptive statistics was used to analyse categorical variables and statistical analysis was done using the Chi-Square Test. Statistical significance was set at p<0.05. Full ethical approval for this study was obtained prior to patient recruitment and data collection.

## Results

There was a total of 240 participants with a majority of males (85.4%) and slightly more than half were aged between 18 to 30 (50.4%), with a mean of 31.05±9.211. They were made up of 70% Malays, 20.8% Indians, 7.5% Chinese and 1.7% others. Most of the participants received nuclear family support (47.9%), followed by joint family support (41.3%) and 10.8% has no familial support. Additionally, 82.5% of them were employed at the time of the study, while students and unemployed participants each made up of 8.8% of the respondents. Most of the respondents were victims of motor vehicle accidents (MVA) (71.7%) and the remainders made up of victims of car or lorry accidents, fall, occupational accident, domestic violence and sports injury. A total of 89.6% of the respondents sustained fractures following the trauma. Other injuries include dislocation (4.6%), ligament rupture (5%) and others (0.8%).

A majority of participants experienced severe pain at the scene of trauma, with a mean score of 7.94±2.071. Meanwhile, 15.8% and 5% of participants reported experiencing moderate and minor pain on the scene, respectively. More than three-quarters of our participants (77.9%) sustained moderate injuries, as indicated by the Injury Severity Score (ISS), with a mean severity score of 11.02±4.232. Additionally, 4.2% had minor injuries, 16.3% had serious injuries, and 1.7% had severe injuries.

We determined using the stipulated questionnaire that our participants who developed PTSD were 12.9% with 95% confidence interval. Similarly, 43.4% of the participants developed depression symptoms post-trauma with 95% confidence interval was computed at between 1.54–1.78, with the mean value of 1.66.

The comparison data were computed using Chi-square (p-value) tests. All of the demographic factors had no significant association to the prevalence of PTSD symptoms (p>0.05) (Table I). All of the demographic factors had no significant association to the prevalence of depression symptoms (p>0.05) (Table II).

**Table I:** Cross tabulation of demographic factors with prevalence of post-traumatic stress disorder (PTSD) symptoms.

Demographics		Categories		PTSD		P-value
		n	%	n	%	
Gender	Female	32	15.3	3	9.7	0.587
	Male	177	84.7	28	90.3	
Age	18-30	105	50.2	16	51.6	0.854
	31-45	84	40.2	13	41.9	
	Above 45	20	9.6	2	6.5	
Ethnicity	Malay	145	69.4	23	74.2	0.732
	Indian	44	21.0	6	19.4	
	Chinese	16	7.7	2	6.4	
	Others	4	1.9	0	0.0	
Familial Support	Joint Family	92	44.0	7	22.6	0.050
	Nuclear Family	94	45.0	21	67.7	
	Nuclear Family	23	11.0	3	9.7	
Employment Status	Student	20	9.6	1	3.2	0.159
	Employed	169	80.9	29	93.6	
	Unemployed	20	9.5	1	3.2	
Types of Trauma	Motor Vehicle Accident (MVA)	154	73.7	18	58.0	0.536
	Car / Lorry Accident	14	6.7	4	12.9	
	Fall	21	10.0	5	16.1	
	Occupational Accident	13	6.2	2	6.5	
	Domestic Violence	1	0.5	0	0.0	
	Sports Injury	6	2.9	2	6.5	
Mechanism of Injury	Bone Fracture	189	90.4	26	83.8	0.549
	Dislocation	9	4.3	2	6.5	
	Ligament Rupture	10	4.8	2	6.5	
	Others	1	0.5	1	3.2	

**Table II:** Cross tabulation of demographic factors with prevalence of depression symptoms.

Demographics	Categories	Depression				
		Absent		Present		P-value
		n	%	n	%	
Gender	Female	18	13.2	17	16.3	0.499
	Male	118	86.8	87	83.7	
Age	18-30	66	48.5	55	52.9	0.723
	31-45	58	42.7	39	37.5	
	Above 45	12	8.8	10	9.6	
Ethnicity	Malay	97	71.3	71	68.3	0.403
	Indian	24	17.7	26	25.0	
	Chinese	12	8.8	6	5.8	
	Others	3	2.2	1	0.9	
Familial Support	Joint Family	63	46.3	36	34.6	0.102
	Nuclear Family	57	41.9	58	55.8	
	No Support	16	11.8	10	9.6	
Employment Status	Student	12	8.8	9	8.7	0.918
	Employed	113	83.1	85	81.7	
	Unemployed	11	8.1	10	9.6	
Types of Trauma	Motor Vehicle Accident (MVA)	107	78.7	83	79.9	0.951
	Fall	16	11.8	10	9.6	
	Occupational Accident	8	5.9	7	6.7	
	Domestic Violence	1	0.7	0	0.0	
	Sports Injury	4	2.9	4	3.8	
Mechanism of Injury	Bone Fracture	120	88.2	95	91.4	0.844
	Dislocation	7	5.2	4	3.8	
	Ligament Rupture	8	5.9	4	3.8	
	Others	1	0.7	1	1.0	

We then computed the comparison data using Chi-square (p-value) tests and found that pain level and the severity of the injuries has no significant association to the prevalence of PTSD symptoms (p>0.05) (Table III). Similarly, we found that the degree of pain experienced, and the severity of the injuries also had no significant association to the prevalence of depression symptoms (p>0.05) (Table IV).

**Table III:** Cross tabulation of pain and injury severity with prevalence of post-traumatic stress disorder (PTSD) symptoms.

Severity	Categories	Post-Traumatic Stress Disorder (PTSD)				P-value
		Unlikely		Likely		
		n	%	n	%	
Pain Score	Minor	11	5.3	1	3.2	0.195
	Moderate	36	17.2	2	6.5	
	Severe	162	77.5	28	90.3	
Injury Severity Score (ISS)	Minor	7	3.4	3	9.7	0.127
	Moderate	167	79.9	20	64.5	
	Serious Severe	31 4	14.8 1.9	8 0	25.8 0.0	

**Table IV:** Cross tabulation of severity with prevalence of depression symptoms.

Severity	Categories	Depression				P-value
		Absent		Present		
		n	%	n	%	
Pain Score	Minor	8	5.9	4	3.9	0.191
	Moderate	26	19.1	12	11.5	
	Severe	102	75.0	88	84.6	
Injury Severity Score (ISS)	Minor	4	2.9	6	5.8	0.133
	Moderate	113	83.1	74	71.2	
	Serious	18	13.2	21	20.2	
	Severe	1	0.8	3	2.8	

However, we determined that PTSD had a significant association to the prevalence of depression symptoms. The results showed that most of the participants with probable PTSD had associated depression (93.5%) with a p-value of less than 0.001 (Table V).

**Table V:** Cross tabulation of the prevalence of post-traumatic stress disorder (PTSD) symptoms with the prevalence of depression symptoms.

Severity	Categories	Post-Traumatic Stress Disorder (PTSD)				P-value
		Unlikely		Likely		
		n	%	n	%	
Depression	Absent	134	64.1	2	6.5	<0.001
	Present	75	35.9	29	93.5	

## Discussion

Orthopaedic post-trauma patients are at risk of developing post-traumatic stress disorder (PTSD) and depression as a result of their physical injuries and the psychological impact of the traumatic event. According to our results however, most of our participants were unlikely to have PTSD. The 95% confidence interval was computed to be 1.09–1.17, with a mean value of 1.13. The fact that the confidence interval is narrow shows that our estimate of the mean is relatively precise, indicating that we can be fairly confident that the true population mean is close to the sample mean of 1.13. This suggests that the prevalence of PTSD in orthopaedic post-trauma patients is relatively low, with only a small proportion of patients exhibiting symptoms of the disorder. Additionally, the Chi-square (p-value) tests revealed that none of the demographic factors had a significant association with the prevalence of PTSD symptoms (p>0.05).

Similarly, most of the participants (56.7%) did not develop depression post-trauma either, with a 95% confidence interval computed to be 1.54–1.78 and a mean value of 1.66. This indicates that the prevalence of depression among orthopaedic post-trauma patients is relatively moderate, with slightly less than half of the patients experiencing symptoms of depression such as persistent feelings of sadness, anxiety or emptiness, loss of interest or pleasure in activities once enjoyed, insomnia, fatigue or loss of energy, changes in appetite and/or weight, and feelings of worthlessness or guilt. The Chi-square (p-value) tests also indicated that none of the demographic factors had a significant association with the prevalence of depression symptoms (p>0.05).

From this, we concluded that the prevalence of PTSD and depression among our orthopaedic post-trauma patients is low to moderate, with a small proportion of patients exhibiting symptoms of PTSD and slightly more than half experiencing symptoms of depression. Additionally, none of the demographic factors were significantly associated with the prevalence of depression symptoms. According to a systematic review and meta-analysis published in the Journal of Orthopaedic Trauma in 2020, the authors found that the prevalence of PTSD symptoms among orthopaedic trauma patients ranged between 1–45%, with a pooled prevalence estimate of 17%, while the prevalence of depression symptoms amongst these patients range from 3% to 80%, with a pooled prevalence estimate of 36^12^. Overall, the study suggests that PTSD and depression are not uncommon among orthopaedic post-trauma patients hence the importance of screening for and addressing these mental health issues, similar to our own findings.

The definition of "traumatic" in the DSM-5 has been clarified and condensed, and it now refers to situations that involve “actual or threatened death, serious injury, or sexual violence”. One of the most common psychological effects of injuries is PTSD. The negative core schemas of a person, such as "I'm worthless," "I'm unlovable," and others, may be influenced by cognitive processing in the posttraumatic situation, which also leads to the development of PTSD. Another possible effect of injuries is depression, which can be exacerbated by this. Other research findings have shown that the severity of physical injury is unrelated to the prevalence or severity of postinjury psychological repercussions^13^. Results from our study reinforces this finding as evidenced by the results from the Chi-square tests conducted which revealed no significant association between the severity of injuries sustained and the prevalence of PTSD symptoms (p>0.05) or symptoms of depression (p>0.05). This highlights the need for early intervention in all patients who have sustained trauma, regardless of the severity as even mild injuries can result in traumatic stress reactions. Models for early intervention have been created to lessen the negative effects of potentially traumatic incidents. In order to focus interventions on the patients who need them the most, these models require accurate predictors of risk for the emergence of psychological effects. Finding the post-traumatic patients who are most likely to have subsequent depression or PTSD is difficult. There may be observable risk factors for the emergence of postinjury psychological problems, according to studies conducted in the last ten years. Acute stress symptoms, prior depression treatment, prior trauma exposures, a lack of financial and social resources, a history of maladaptive coping mechanisms, concerns about injury, and an assessment of acute stress reactions are all factors that are linked to an increased likelihood of developing depression or PTSD^14^.

According to our results, most of our participants developed post-traumatic stress disorder (PTSD) associated with depression, compared to either developing PTSD or depression alone. The Chi-square test showed that PTSD has a significant association with the prevalence of depression symptoms (p<0.001). This showed that orthopaedic post-trauma patients who develop either PTSD or depression only are relatively low. There are some reasons that might explain the co-occurrence of PTSD and depression symptoms: both conditions share common symptoms which are listed in PCL-5 and PHQ-8. Both PTSD and depression can cause one to feel sadness, hopelessness, depress, and despair. Individual with PTSD may also experience guilt related to the traumatic event which can later contribute to the development of depression^14,15^. Additionally, both PTSD and depression can lead to social isolation and difficulty with relationships, which can further exacerbate symptoms of both conditions.

Viewing from a neurobiological pathway, both post-traumatic stress disorder (PTSD) and depression symptoms are involved in the hypothalamic pituitary adrenal (HPA) axis and serotonergic system. The HPA axis is a vital regulator of the stress response. In response to stress, the hypothalamus releases corticotropin-releasing hormone (CRH), which stimulates the pituitary gland to release adrenocorticotropic hormone (ACTH). ACTH then triggers the release of cortisol from the adrenal cortex, allowing the body to mobilise energy and cope with stress. In individuals with PTSD and depression, the HPA axis is often dysregulated. It is this dysregulation of the HPA axis that is linked with symptoms of both PTSD and depression^16^. Serotonin is a neurotransmitter that plays a role in regulating mood, appetite, and sleep. Studies have shown that individuals with PTSD and depression have decreased serotonin activity, which is associated with symptoms such as anxiety, irritability, and sleep disturbances^17^. Thus, serotonin reuptake inhibitors (SSRIs), which increase serotonin activity, are commonly used to treat both PTSD and depression.

A study of this nature where focus in placed on the prevalence of PTSD and depression symptoms in post-injured orthopaedics patients is rare in Malaysia. Our findings are similar to other reports published elsewhere^12,18,19^, although pain does not appear to be such a major contributing factor^19^. Using the Beck Depression Inventory (BDI), Crichlow *et al* studied their pool of orthopaedic trauma patients and found evidence of depression approaching 45^20^, close to our own figure of 43.3%. In a systematic review of 17 applicable studies published, Versluijs *et al*^21^ found little or no relationship between injury severity and the occurrence of depression amongst their patient, as did we. Despite the majority of post-trauma patients exhibiting no outward evidence of either PTSD or depressive symptoms, these issues ought to be scrutinised and adequately addressed so that at-risk patients are not missed (or dismissed). Attending health care professionals ought to ensure that proper physical and emotional counselling are always available to these cohort of patients thus establishing a more holistic recovery^22,23^.

One of the limitations of this study is a small sample size. However, despite the small number of participants, we believe that the results remain significant with respect to the occurrence of PTSD and depression symptoms amongst post-trauma patients.

## Conclusion

Only a few of the participants are likely to develop post-traumatic stress disorder (PTSD) while close to half of the participants are likely to have developed depression in this study. Neither the prevalence of PTSD nor depression are associated with the demographics or the severity of injury. Furthermore, it was also shown that most participants with probable PTSD have associated depression symptoms also. Regardless, it is highly suggested that doctors involved in skeletal trauma patient care should adequately screen each patient in order to detect early symptoms of PTSD and depression and treat them accordingly.

## Ethical Approval

This study was conducted in compliance with ethical principles outlined in the Declaration of Helsinki and Malaysian Good Clinical Practice Guideline and received ethical approval from the International Medical University Joint Committee (IMUJC) as well as the National Medical Research Registry (NMRR) and Malaysian Medical Research Ethics Committee (MREC).
